# A Consumer Segmentation Study for Meat and Meat Alternatives in Switzerland

**DOI:** 10.3390/foods10061273

**Published:** 2021-06-03

**Authors:** Franziska Götze, Thomas A. Brunner

**Affiliations:** School of Agricultural, Forest and Food Sciences HAFL, Bern University of Applied Sciences BFH, Länggasse 85, 3052 Zollikofen, Switzerland; thomas.brunner@bfh.ch

**Keywords:** meat consumption, meat alternatives, market segmentation, cluster analysis, Switzerland

## Abstract

The aim of this study was to identify consumer groups regarding meat and meat alternatives, which are homogeneous in themselves but very different from one another. To date, the literature has analysed the attitudes towards, and the motives behind, the consumption of meat and meat alternatives. However, segmentation research portraying homogeneous consumer groups that are consuming or willing to consume meat alternatives is lacking. This study closes this research gap and, in doing so, also shows how meat consumption is related to the consumption of alternative products. A questionnaire was sent out to a random sample in the German- and French-speaking parts of Switzerland, resulting in 561 responses. A hierarchical cluster analysis using seven scales revealed six distinct consumer groups, which covered all types of consumers, from the uncompromising meat-eater to the health-conscious meat avoider. The results show that meat alternatives are not always consumed as a substitute for meat but can also be a complementary component in one’s diet. This study contributes to the scientific literature by providing useful information for the food industry involved in producing and marketing meat and meat alternatives to different target groups.

## 1. Introduction

In recent years, vegan and vegetarian diets, including plant-based sources of protein, have become increasingly appealing to consumers [1,2]. As of 2020, according to Swissveg, the largest representation of vegetarians and vegans living in Switzerland, approximately 5% of the Swiss population eats a vegetarian or vegan diet, and including flexitarians (i.e., occasional meat consumers), the population makes up nearly a quarter of the Swiss population. (https://www.swissveg.ch/veg-umfrage (in German and French) accessed on 11 May 2021) The reasons for following a meat-free diet are diverse. Whereas a diet without meat is regarded as healthy, high meat consumption is associated with modern lifestyle diseases, such as cardiovascular diseases and cancer [3,4,5]. In addition, at least some consumers are consciously avoiding meat and meat products due to an increased awareness for sustainability, given the growing realisation that meat production is “the most impacting activity in food production” [5,6] (p. 1255). Ethical considerations, such as animal welfare, are also becoming increasingly important in the consumption decisions of consumers in industrialised countries [2,7,8].

As a small but growing number of consumers are willing to reduce meat consumption, avoid meat completely or replace it with (for example) plant-based alternatives, the food industry has reacted positively to this growing trend. Today, a decent range of alternative products is already available in the market and is expected to grow in the future [2,4,8,9,10]. While the consumption of meat and meat products in Switzerland has remained constant throughout the last decade (48–52 kg/person/year) [11], a growing number of alternatives to meat can be found in the food market. According to Guigoz [12], a quarter of the protein consumed in Switzerland was of plant origin as of 2011. Hence, on the one hand, a part of the population seems to have reduced meat in their diet, and on the other, the remnant appears to be consuming higher amounts of meat over time.

Despite the growing range of meat alternatives present in the market, their sales remain low to date. (In the US, for example, sales of meat alternatives in 2019 rose by 18% (compared to 2018) to almost $939 million (https://www.gfi.org/marketresearch (accessed on 11 May 2021)) However, forecasts hint that the market for plant-based meat alternatives will develop further in the coming years and that the demand for such products will increase [10,13]. Clustering consumers into groups that are homogeneous in themselves but are as unique as possible from one another is not an easy task, as such consumption decisions are complex, and many factors may play a role in the overall outcome. Segmentation of the market for meat alternatives can help to improve our understanding of the needs of individual consumer groups. A better understanding of the nature of today’s consumers of meat and meat alternatives, as well as their consumption motives and needs, and the segments that might consume or avoid meat alternatives in the future is, moreover, interesting for those producing and selling meat alternatives. This is especially true of Swiss producers, as the preference for domestic products is typically high in Switzerland [14]. Hence, this study aims to portray the different types of consumers with regard to their attitudes towards meat and meat alternatives and their consumption behaviours. It will also identify the relationship between the consumption of meat and meat alternatives.

## 2. Materials and Methods

### 2.1. Database and Sample

A questionnaire entitled “Consumption of meat and meat alternatives” was sent out via mail to an extensive sample of households in the German- and French-speaking parts of Switzerland (in German and French, respectively). The term “meat alternative” was defined as follows in the questionnaire: “a food product you consciously consume instead of a piece of meat”. We left this definition somewhat open intentionally to avoid excluding any food products. The sample was randomly selected from the Swiss telephone directory. The whole sample comprised 4000 addresses, of which 184 were returned unanswered, as the addressees were deceased (11), had moved to a new address (67), or were unable to answer for various reasons (106). The questionnaire was sent out on 12 September 2016, and the responses were collected from 16 September until 2 November 2016. In addition, 18 individuals refused to accept the letter and 22 questionnaires were returned as is (i.e., the addressees sent back the blank questionnaire, cover letter, and return envelope in another envelope). Hence, 3776 (4000 min 224) households finally received the questionnaire, of which 632 returned completed questionnaires. The remaining 3114 households did not return the questionnaire. The initial number of 632 respondents was further reduced due to a check item included in the questionnaire. To ensure the validity of the data, the respondents were asked to mark a certain box on the six-point Likert scale. (The exact wording was “...to ensure the data quality, please tick the second box here”.) To ensure that the quality of the data collected met the requirements of this study those who did not mark the correct box were excluded (45). Another 26 questionnaires were disregarded because the respondents did not answer 40 or more questions. Thus, the total number of completed questionnaires considered in the analysis amounted to 561.

### 2.2. Segmentation Procedure

The clustering process was based on a set of seven scales. These scales are displayed in Table 1 and comprise between two and five items. Several similar items were queried to increase the accuracies of the answers. The scales were sourced partly from past studies. As this is an explorative study, several new items and scales were added to accurately capture consumer behaviour. The new scales were tested using principal component analysis with varimax rotation using Kaiser normalisation.

Each respondent’s agreement with each item was queried using a six-point Likert scale. This scale (without a middle, i.e., neutral, response option) was consciously chosen to classify the respondents as clearly as possible. A reliability analysis was performed to test the internal consistency of each scale. One item (“Eating lots of meat is unhealthy”) was removed from the scale *Contra meat* to reach a satisfactory Cronbach’s α (the average of all possible correlations among the items to be included in the scale). Even though it can be assumed that the unhealthfulness of eating large amounts of meat is a strong argument against meat consumption, the item showed a low correlation to the other two items of the scale. After its removal, Cronbach’s α increased from 0.51 to 0.61 for this scale, indicating sufficient reliability. The reliability measures for the seven scales in Table 1 range from 0.61 to 0.93.

To cluster consumers in terms of their attitude towards meat and meat alternatives and their consumption, a hierarchical cluster analysis was carried out using the seven scales displayed in Table 1. The cluster analysis included 548 respondents, namely the households that answered the questionnaire in entirety with regard to the items displayed in Table 1. Ward’s method was used for the cluster analysis, and the squared Euclidean distance served as the distance measure. While applying Ward’s method, we did not merge the objects that show the smallest distance, instead those that increased the error sum of the squares (the variance criterion being the sum of the squared distances between the identified clusters to the cluster centroid) the least. (Backhaus et al. [18] stated that Ward’s method can be regarded as a “very good merging algorithm”. To compare the results, the K-means clustering method was tested further. As this approach resulted in very similar clusters, Ward’s method was selected for this work.) Next, the agglomeration schedule for cluster solutions of 10 to 2 clusters were analysed and the relative changes in the error sums of the squares of each merging step were compared as the number of clusters decreased. As the relative change in the coefficient is typically large from the two- to the one-cluster solution [18], this option was not considered. Large changes in the coefficients from one cluster solution to the next imply that two heterogeneous clusters have just been merged (which is not what is desired). Therefore, the number of clusters that exist immediately before such a substantial increase occurs was of interest. After applying this procedure, solutions of five and six clusters were further examined.

**Figure 1:**
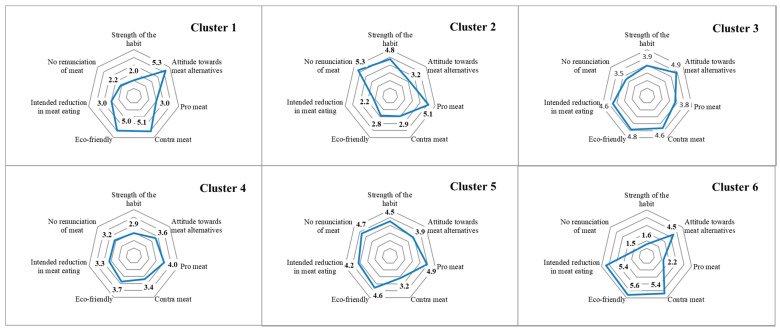
Graphical representations of the six identified clusters for the clustering scales and their means. For the items of all the scales, the agreement was queried as follows: 1—Do not agree at all, 6—Strongly agree.

Both cluster solutions led to significant differences among all cluster variables (as revealed by analysis of variance with contrast tests). For both cluster solutions, significant differences were found among clusters for almost all scales. To determine the best possible solution, the five- and six-cluster solutions were then plotted using the means of the segmentation scales (Figure 1).

As shown in Figure 1, the six-cluster solution leads to obviously different clusters. These differences could be approved for almost all scales and clusters, as mentioned above.

## 3. Results

The hierarchical cluster analysis and following analyses of variance revealed six distinct clusters: the environmentally and health-oriented meat-eaters (Cluster 1—16.8% of the survey participants), the uncompromising meat-eaters (Cluster 2—18.1%), the moderate meat-eaters who are willing to replace meat (Cluster 3—15.9%), the indifferent but moderate meat-eaters (Cluster 4—21.2%), the environmentally-conscious regular meat-eaters (Cluster 5—13.7%) and the environmentally and health-conscious meat avoiders (Cluster 6—14.4%) (Figure 1 and Table 2). The clusters were further analysed with regard to their sociodemographic profiles and other characteristics. Analyses of variance (with contrast tests) were carried out to evaluate whether each cluster significantly differed from the other clusters in terms of the characteristics presented in Table 3, Table 4, Table 5, Table 6 and Table 7.

### 3.1. Cluster 1: Environmentally and Health-Oriented Meat-Eaters

The typical environmentally and health-oriented meat-eater does not consume too much meat compared to the respondents in the other clusters (Table 5). Most consumers in this cluster, however, have eaten and bought meat alternatives in the past and are the second-highest respondents in terms of the average consumption of these alternatives (Table 5). Further, their overall diet is hardly driven by habits (Table 4). It can be assumed that the subjects in this cluster will not necessarily change their consumption patterns; these consumers already eat a comparatively small amount of meat and are not willing to change this habit, as indicated by the low scores (i.e., indicating rejection) for the scales *Intended reduction in meat-eating* and *No renunciation of meat* (Table 1 and Figure 1). Apparently, the average consumer in this cluster finds his or her meat consumption acceptable and wants to retain the status quo. However—and possibly due to the comparatively low meat intake—these consumers might want to add more meat alternatives to their diet than they currently consume (on average, 1.7 portions per week; Table 5). According to the findings, consumers in this cluster are of much interest to producers and marketers of meat alternatives because of their exceedingly positive attitude towards and their second-highest consumption rate of such products (Figure 1 and Table 5). The reasons for their high affinity towards meat alternatives are, among other factors, related to health and environmental concerns. These consumers gauge meat consumption as rather negative and recognise the environmental disadvantages of meat production and consumption (Figure 1). Furthermore, the subjects in this cluster are not very price-sensitive—in fact, it is the least price-sensitive of all the clusters (Table 4). Hence, it is not surprising that their agreement with the arguments in favour of meat alternatives is rather high (Table 6), whereas that with the contra arguments is rather low (Table 7).

Regarding the sociodemographic profile, the typical consumer in this cluster tends to be female, is equipped with a solid knowledge about food and nutrition and is characterised as earning an average income (Table 3).

### 3.2. Cluster 2: Uncompromising Meat-Eaters

The typical consumer in the cluster of uncompromising meat-eaters differs markedly from a consumer in Cluster 1. The uncompromising meat-eater is convinced about the correctness of his or her meat consumption and thus exhibits a strong meat-eating habit (Figure 1). It can be assumed that he or she eats meat regularly out of habit, and the average amount of meat consumed per week is the highest across all the clusters (Table 5). Therefore, it seems unlikely that such a consumer will give up meat consumption completely. He or she is not willing to reduce or forego it, does not seem to be aware of the environmental impacts of meat production and does not perceive meat consumption in a negative way (Figure 1). Due to the high affinity towards eating meat, his or her low agreement with the arguments in favour of meat alternatives (Table 6) and high agreement with the contra arguments (Table 7), it is not surprising that this consumer does not have a positive attitude towards meat alternatives (Figure 1). For the subjects in this cluster, meat is part of a balanced diet, and the taste of meat alternatives just does not compare to that of real meat (Table 7). The latter notion might also stem from the fact that their experience with such products is rather low—in fact, just over half of the respondents in this cluster have ever eaten meat alternatives and as few as a quarter have ever purchased them (Table 5). Hence, the subjects in this cluster are least likely out of all the clusters to purchase meat alternatives.

Regarding the sociodemographic profile, the highest share of men appears in this cluster (Table 3). Moreover, the respondents in this cluster know less about food and nutrition than the average for the overall sample. The subjects in this cluster also exhibit a slightly higher body mass index (BMI), which might partly result from the higher share of men in it. Table 4 further confirms that the average consumer in this cluster is not only a habitual meat-eater (given the highest amount of meat consumed; Table 5), but also follows his or her habits and routine regarding his or her overall diet. Furthermore, ethical considerations are less important to the subjects in this cluster (however, on a high level), and these respondents tend to shop to a lesser extent for organic food (Table 4). Even though many criteria, including regionality, are not likely to be important to this type of consumer, the Swiss origin of the product (*Swissness*) does seem to be important; the second-highest affinity towards Swiss products appears in this cluster (Table 4).

### 3.3. Cluster 3: Moderate Meat-Eaters Who Are Willing to Replace Meat

This cluster exhibits the meat-eating habit to an extent, but it is not as strong as that of the uncompromising meat-eaters (Cluster 2; Figure 1). The average moderate meat-eater is, however, and in contrast to those in Clusters 1 and 2, willing to reduce his or her meat consumption (Figure 1). These consumers think that meat production is not beneficial for the environment and consider eating meat as rather unhealthy (*Contra meat*; Figure 1). Regarding their food-related attitudes and preferences, the respondents in this cluster are fairly similar to the average; the analyses of variance did not reveal significant differences from the subjects in the other clusters in terms of the scale means (Table 4). With regard to their consumption behaviour, while these consumers do not eat more or less meat than the average, they do consume slightly lower amounts of meat alternatives (Table 5). Finally, the consumers in this cluster agree with both the pro and contra arguments pertaining to meat alternatives. Whereas these consumers clearly recognise the advantages of such products in terms of animal welfare and sustainability and agree with the fact that the population eats too much meat anyway (Table 6), on the whole, they also feel that meat alternatives cannot really replace meat, do not taste like meat and are not as good or necessary to include in the diet as meat (Table 7).

Significant differences were observed regarding the sociodemographic characteristics in terms of the residential area and education (Table 3). The consumers in this cluster are more likely to live in agglomerations or rural areas than a city. Furthermore, this cluster (together with Cluster 6) is characterised by the highest average level of education among all the clusters.

### 3.4. Cluster 4: Indifferent But Moderate Meat-Eaters

The typical consumer in this cluster is a moderate meat-eater and is indifferent about most food-related issues (Table 4 and Table 5). Figure 1 shows neutral mean scores for most clustering scales. This means that the respondents of this cluster neither approved of nor rejected most of the items. The average consumer seems to be undecided about whether he or she wants to reduce meat consumption or is simply indifferent. Moreover, this cluster is not characterised by strong meat-eating habits (Figure 1). Note, however, that this does not necessarily mean that these consumers do not eat meat at all (Table 5). Rather, the frequencies and amounts of meat consumption may fluctuate over time. Meat consumption is perceived as rather healthy; however, the average consumer in this cluster does not reject (and hence is neutral about the notion) the arguments against meat consumption (Figure 1). Furthermore, this consumer appears to be undecided about meat alternatives. He or she might also be indifferent to them (i.e., shows neither a positive nor a negative attitude). Regarding food in general, a high affinity (albeit the lowest across all clusters; Table 4) was noted towards natural and unprocessed food. Moreover, ethical considerations matter to these respondents (to a lesser extent than that for the other consumer groups, however). It can also be assumed that organic food is consumed less often, as it is less important to the consumers in this cluster than to those in the other clusters. However, regionality matters, although less than in the other clusters.

The typical consumer in this cluster is less educated about nutrition than those in the other clusters, which can be evidenced by the second-lowest score for knowledge (Table 3).

### 3.5. Cluster 5: Environmentally Conscious Regular Meat-Eaters

The regular meat-eater in this cluster is aware of the environmental effects of producing and consuming meat (*Eco-friendly*; Figure 1). This consumer eats meat products on a rather regular basis, in part because he or she perceives meat consumption as positive (*Pro meat*; Figure 1). Whether the subjects in this cluster want to reduce their meat consumption is unclear, because the habit of eating meat is quite strong and the willingness to reduce or forgo meat consumption is low. Notably, the attitude towards meat alternatives is rather positive (Figure 1). Meat alternatives could therefore be considered more as a complementary product than as an alternative for these consumers.

In general, these consumers are mindful about the sensory qualities of food (Table 4). Their sensory enjoyment is more important than in the case of the respondents in the other clusters. Furthermore, they like to cook. Habits are also important—for food in general and even more so than for meat alone. In addition, these consumers are rather price-sensitive even though they prefer products from their own country (which, in the Swiss case, are often more expensive than imported ones; Table 4). A good price/performance ratio also matters to them, hence, good value for money determines which items find their way into the shopping baskets of environmentally conscious regular meat-eaters. Therefore, it is also not surprising that organic food is not more important than average compared to the cases of the other clusters, as it is often more expensive than conventional food. Regarding meat alternatives, around two-thirds have already eaten them, but less than half of the cluster has bought meat alternatives in the past (Table 5). Hence, the majority have at least some experience with these products. Nevertheless, meat consumption is still high (the third highest of all the clusters) and the consumption of meat alternatives is low—the second-lowest, with only 50 g consumed on average per week. Despite the strong habit and rather high meat consumption (Figure 1 and Table 5), the environmentally conscious regular meat-eater agrees with some of the arguments in favour of meat alternatives (Table 6): Swiss society’s meat consumption is too high and less animals have to die when meat alternatives are eaten instead of meat. However—and this likely explains the low consumption of meat alternatives—the existing alternatives are not to the taste and preferences of this cluster; they do not taste good (enough) and are not really replacements for meat (Table 7). Finally, these respondents strongly believe that meat is part of a balanced diet and it is possible to eat meat-free meals without consuming meat alternatives.

The sociodemographic profile and other characteristics of the respondents in this cluster are not much different from those in the other clusters (Table 3). The average person in this cluster earns a slightly higher income than the overall average.

### 3.6. Cluster 6: Environmentally and Health-Conscious Meat Avoiders

The environmentally and health-conscious meat avoiders are the most stringent regarding meat consumption (Figure 1 and Table 5). They are not habituated to meat consumption, and thus, it can be assumed that these consumers are willing to reduce meat consumption over time, perhaps giving it up even completely (Figure 1). Therefore, vegetarians and vegans can be expected to exist in this cluster. Their reasons for reducing meat or avoiding it completely are related to the perceived unhealthfulness of meat (*Contra meat*), the environmental side effects of meat consumption and production (Figure 1), as well as the wish to consume ethically correct foods (i.e., pay heed to animal welfare issues) (Table 4). These consumers—similar to those in Clusters 1 and 3—are very likely to purchase meat alternatives, as they have a positive attitude towards them (Figure 1), and nearly all of them have already eaten meat alternatives (82% purchased them; Table 5). Since their habit of meat consumption is weak to nonexistent, it is not surprising that the consumers in this cluster consume the least meat of those in all the clusters. Moreover, as their attitude towards meat alternatives is positive (Figure 1) and they agree with the arguments in favour of meat alternatives (Table 6), it is unsurprising that their consumption of these products is comparatively high—they consume significantly more than those in the other clusters (Table 5).

Regarding food consumption in general, healthfulness is one of the most important decision criteria for this cluster (Table 4) compared to the other clusters. It can be assumed that the food products bought by this cluster are natural (unprocessed), helping these consumers feel good (about themselves) and boost their mood. Furthermore, the propensity of buying organic products is the highest in this cluster. These consumers consider the product origin and prefer products that have been produced in their own region. Finally, this cluster is predominantly comprised of females with above-average knowledge about nutrition (Table 3).

## 4. Discussion

This study strives to contribute to the existing scientific findings regarding the consumption of meat and meat alternatives. Several studies related to the topic of meat substitutes have already been conducted. These studies concern the market and demand for, as well as the consumer acceptance of, meat substitutes. Hoek et al. [22,23,24], for example, analysed how consumers in the UK and the Netherlands perceived meat substitutes, and revealed person- and product-related factors associated with the acceptance of such alternatives. Janssen et al. [25] studied German consumers and showed that the motives to not eat meat are diverse. The present study reveals diverse aspects that can be important for the consumption of meat as well as alternative products. The results of this study suggest that a complex decision-making process exists behind the consumption of meat and meat alternatives. The results of this study are in line with those of Janssen et al. [25] in the sense that different combinations of motives and behaviours—depending on the type of consumer—exist with regard to the consumption of meat and meat alternatives.

Hoek et al. [23,24] and Schösler et al. [26] showed that the consumption of meat substitutes is related to consumers’ familiarity with these products. Interestingly, heavy users of meat substitutes do not necessarily demand substitutes that have a similar appearance to real meat products (e.g., sausages or mince). By contrast, consumers who do not consume meat substitutes as often or regularly tend to want them to look similar to real meat. Accordingly, Hoek et al. [24] also revealed that if meat substitutes have a resemblance to real meat, they have an advantage in the market. This does not necessarily translate to an appearance similar to that of a meat product. The resemblance can also refer to a similar use in meals. Thus, it can be inferred that Cluster 2 (comprising uncompromising meat-eaters) is more likely to consider meat alternatives that are similar to meat in, for example, appearance and uses in meals. Further, it is likely that the subjects in Cluster 6, who are more familiar with meat alternatives than those in Cluster 2, are more likely to consider meat alternatives that are dissimilar to meat.

Furthermore, Hoek et al. [22] showed that for some consumers, the liking for meat substitutes increases over time, suggesting that consumption habits and familiarity with such rather novel products are important. Hence, the more often they eat the product, the more they like it. Cluster 2, with its average of 0.2 servings of meat alternatives per week, is likely to have correspondingly little familiarity (45% have never tried meat alternatives) and a weak habit of eating such products. In contrast, Cluster 6, with its higher weekly consumption and better attitude towards these products, confirms Hoek et al.’s [22] findings. Two Dutch studies from 2012 and 2014 [26,27] found that the consumption of and attitude towards meatless meals not only depends on how familiar one is with meat alternatives, but also on one’s preparation skills for meals containing alternatives or willingness to learn how to prepare such meals. Here, it can be assumed that the respondents who regularly consume meat alternatives (Clusters 1 and 6) also exhibit better cooking skills. For the less habitual consumers (Clusters 2–5), this could be an opportunity to (further) improve their attitude towards these products. In any case, however, all the clusters have a positive attitude towards cooking, and thus, it is certainly worth leveraging on this finding in marketing by, for example, printing recipe ideas on the product packaging.

Janssen et al. [25] identified a cluster that eats a vegan diet as being mindful about animal welfare, environmental and self-related considerations (well-being and health). This cluster is similar to Clusters 1 and 6 in this study. These types of consumers have a weak or almost no habit of eating meat, and those in Cluster 6 would even be willing to further reduce their meat consumption. Similar to Janssen et al. [25], this study reported a cluster that does consider animal welfare issues. However, the environmental and possible health-related consequences of meat consumption are considered to be less important. The difference between the two is that some characteristics of the subjects in Cluster 2 in this work are completely opposite to those described by Janssen et al. [25], in that Cluster 2 is comprised of uncompromising meat-eaters and does not contain vegans. Whereas it is not surprising that vegans avoid meat because of ethical considerations (amongst others), it is less likely that animal welfare matters as much to uncompromising meat-eaters. This is in line with the findings of the Belgian study of De Backer and Hudders [7] that showed that vegetarians and moderate meat-eaters (flexitarians) are concerned about animal welfare.

The results of this study also show that meat alternatives are not necessarily only consumed as substitutes. This study found different types and combinations of meat and meat alternatives consumption, ranging from almost only meat consumption to higher rates of alternatives consumption (Table 5). Therefore, not all consumers are willing to replace meat with an alternative product, which is in line with the results of Onwezen and van der Weele [28] and Graça et al. [29]. However, this study shows that most consumers are positive, or at least not negative (i.e., neutral or undecided) about such products. Moreover, most consumer habits can be said to lie between meat avoidance and uncompromising meat consumption, which was also the case for Graça et al. [29], who identified three clusters of consumers with regard to reduced meat consumption. They also found that apart from a cluster that was unwilling to change its eating habits regarding meat and one that already avoids eating meat because of their disgust for meat and their moral reasons, the biggest cluster contained consumers that lay in between the above two lines of thought. This “in-between” cluster might change its meat consumption behaviour in the future. The prerequisites favouring this change are, according to Graça et al. [29], a “low affective connection towards meat” [29] (p. 80) and the awareness of the consequences of eating meat; the same can also be assumed for the clusters identified in this work, namely due to the subjects’ awareness of the health and environmental consequences of meat consumption. Therefore, Switzerland can be regarded as a promising market for promoting meat alternatives even though meat consumption is still high. Most consumers seem to be willing to purchase or are at least interested in alternative products.

Not only does this analysis partly confirm the results of other studies, it also shows some differences between sociodemographic groups. Men, for example, tend to be more often uncompromising meat-eaters and are thus less likely to be consumers of meat alternatives than women, as they also have a less positive attitude toward such products. Previous studies (e.g., Graça et al. [29]) also found women to be more likely to consume less or no meat. As low meat consumption is associated with a healthy lifestyle [3,4], it is therefore not surprising that the health-oriented meat consumers are more often women than men in our study.

Several studies have investigated meat substitutes that are not of plant origin. Liu et al. [30] studied the acceptance of lab-grown meat in Chinese consumers. Whereas just over half of those surveyed were willing to accept lab-grown meat as a substitute for meat from farm animals, 10% were “definitely (…) unwilling to try” [30] (p. 1) it. Scepticism about lab-grown meat stems from, among other things, the perception that this type of alternative is unsafe, unnatural and disgusting. Smetana et al. [6] applied a life-cycle assessment on different types of meat substitutes while studying insect-based substitutes and concluded them to be favourable options in terms of the environmental footprint. Since the definition of meat alternatives was consciously left quite open in this study, it cannot be ruled out that lab-grown meat or insect-based products could also be considered as meat alternatives for some consumers. For the consumers in Clusters 3 and 5 especially, lab-grown meat could be interesting, as these subjects are aware of the negative environmental consequences of meat consumption but do not necessarily consider meat consumption to be entirely negative.

Florença et al. [31], who studied Portuguese consumers, showed that they were already aware of the sustainability benefits of such alternatives. Florença et al. [31] and Schösler et al. [26] investigated how insects are perceived by consumers. The former [31] found that the overall perception regarding the nutritional value of insect-based foods is low, which might also stem from their scant experience with these products. Schösler et al. [26] showed that most Dutch consumers still perceive insects rather negatively—whether offered as a whole or in processed form. Although attitudes towards insect-based alternatives were not queried in this study, this aspect would be interesting to explore in the future. Given the willingness of the consumers in Clusters 3, 5 and 6 to reduce meat consumption, their awareness of the negative consequences of meat consumption and their positive attitude toward meat alternatives, they could be potential consumers of insect-based meat alternatives.

It can, however, be assumed that the motives to consume lab-grown meat and insects as meat alternatives differ, at least in part, from those to consume plant-based meat substitutes because of the strong prevailing reservations regarding the consumption of such products. Furthermore, lab-grown meat and insects are not a vegetarian alternative and might, therefore, not be considered as alternatives to meat by certain consumers (e.g., vegans; assumably, the subjects in Cluster 6 in this work).

Surprisingly, the respondents in all the clusters in this study care about the degree to which their food is processed and seek natural and unprocessed products; this is true even for the indifferent meat-eaters (Cluster 4). Hence, in contrast to Onwezen and van der Weele [28] (p. 96), this work did not find a “strategically ignorant” consumer group. Furthermore, the respondents in all the clusters, including the uncompromising meat-eaters, consider ethical values in their food consumption decisions (Cluster 2). While it is not possible to pinpoint how ethical considerations are made with certainty, the high appreciation of Swiss products by the consumers in Cluster 2 suggests that the meat products purchased by those subjects might originate from Swiss farms and are, therefore, considered ethically justifiable. The preference for products from the region was found to be high for all the clusters (even though domestic products in the Swiss case tend to be more expensive than imported ones). This finding is in line with Götze et al.’s [32] study on organic food. This finding might be explained by the fact that Swiss food products are bought, at least in part, due to their perceived high quality.

## 5. Conclusions and Implications

To the best of the authors’ knowledge, this study is the first to provide detailed insights into the types of consumers that are willing to buy or already consume meat alternatives. These results are not only relevant for Switzerland, but also for other European as well as non-European countries. Even if the consumption behaviours and food cultures and preferences are not identical with those of other industrialised countries, the trend towards meat alternatives can be observed in most of them. Hence, the results of this study can serve as a starting point for similar research in other industrialised countries. The hierarchical cluster analysis identified different combinations of consumption of meat and meat alternatives, thereby revealing a variety of consumer types. Accordingly, consumers cannot solely be categorised into meat-eaters and meat avoiders. Although these consumer groups exist, the majority can be found to lie in between meat consumption and avoidance. Hence, most consumers do consume meat, but in different quantities and in combination with alternative products. Therefore, this study not only reveals different habits of consuming meat and meat alternatives, but also pinpoints the various scales of consumer willingness to adopt a diet that includes meat alternatives. The analysis also shows that even though the attitudes towards meat and meat alternatives differ, certain aspects are important to all consumer groups, such as consuming ethically correct and natural foods as well as the domestic nature of the product origin. These arguments could be leveraged more strategically in food marketing.

The results are intended for those producing and marketing meat and meat alternatives, as meat alternatives thus far constitute only a small proportion of the market (although one with potential for growth). The results suggest that both product development and marketing should be more target group-oriented to meet the needs of different target consumer groups. For the food industry, such information about consumer segments can be helpful for consumer-oriented marketing and could raise the success rates of product development. Depending on the aspects that matter to consumers—the study shows how diverse their needs are—the advantages in terms of health and the environment, ethical aspects and the naturalness or origin of the product might be emphasised when marketing meat alternatives.

A limitation of this study is that the average age of its respondents is slightly higher than that of the Swiss adult population (57.4 vs. 49.3 years), which is natural given that the survey targeted the people who are mainly responsible for food purchases in the household. Future studies could investigate a younger target group to complement the results of this research, as young consumers often abstain from eating meat as well. They could, therefore, also be an interesting target group for producers of meat alternatives. In addition, further research is needed in terms of product design. The results are, however, a good starting point for product development activities. This work provides solid evidence that many consumers are not negative about meat alternatives. It is, however, unclear to what extent the existing products correspond to consumer needs in terms of healthfulness, environmental friendliness, price, regionality, similarity or dissimilarity to meat and so on. Further, if they do not meet consumer needs, it is worth exploring how such products should appear and taste. Except for the uncompromising meat-eaters who might not eat meat alternatives in the future, all the other consumer groups, comprising most of the sample in this work, offer at least some, if not great, potential.

## Figures and Tables

**Table 1:** Scales, items (including their source(s)), means, and reliability scores.

Scales and Items	Item Source	Mean Score
*1. Strength of the habit* (Eating meat is something...)		
…I do without thinking.	Verplanken and Orbell [15]	2.69
…that would cost me effort not to do.		3.00
…that is part of my daily/weekly routine.		3.65
…I would find hard not to do.		3.26
Cronbach’s *α* = 0.82		
*2. Attitude towards meat alternatives* (I think meat alternatives are...)		
…bad (1)—good (6)	New	4.10
…not important (1)—important (6)		4.20
…not worth supporting (1)—worth supporting (6)		4.14
…negative (1)—positive (6)		4.43
Cronbach’s *α* = 0.89		
*3. Pro meat*		
Eating meat is healthy.	Almli et al. [16]	3.82
Eating meat is necessary for obtaining beneficial nutrients.		3.53
Meat contains important nutrients.		4.55
Meat is good for general health.		3.65
Meat is an important part of a healthy diet.		3.81
Cronbach’s *α* = 0.93		
*4. Contra meat*		
A meatless diet is worrisome in terms of health.	Verbeke [17]; Almli et al. [16]	3.94
One can easily eat a meatless diet.		4.14
Cronbach’s *α* = 0.61		
*5. Eco-friendly*		
Eating a lot of meat causes environmental damage.	New	4.80
Reducing meat consumption is an effective measure against climate change.		4.11
I try to eat less meat to be environmentally conscious.		4.10
Cronbach’s *α* = 0.78		
*6. Intended reduction in meat eating*		
I would like to reduce my meat consumption.	New	3.37
I would like to eat less meat.		3.37
I try to restrict my meat intake.		4.17
Cronbach’s *α* = 0.80		
*7. No renunciation of meat*		
I would find it difficult to reduce my meat consumption.	New	3.19
Eating meat is an important part of my eating habits.		3.52
I cannot and do not want to imagine a life without meat.		3.51
Cronbach’s *α* = 0.88		

For the items of all the scales except the items of scale 2, namely Attitude towards meat alternatives, the agreement was queried as follows: 1—Do not agree at all, 6—Strongly agree.

**Table 2:** Cluster names and shares.

	Cluster Name	Share (%)
Cluster 1	Environmentally and health-oriented meat-eaters	16.8
Cluster 2	Uncompromising meat-eaters	18.1
Cluster 3	Moderate meat-eaters who are willing to replace meat	15.9
Cluster 4	Indifferent but moderate meat-eaters	21.2
Cluster 5	Environmentally conscious regular meat-eaters	13.7
Cluster 6	Environmentally and health-conscious meat avoiders	14.4

**Table 3:** Comparison of clusters with regard to their sociodemographic profiles.

	Ø	Cluster 1		Cluster 2		Cluster 3		Cluster 4		Cluster 5		Cluster 6	
Share of women ^a^ (%)	65.6	80.4		40.2		63.2		69.0		64.0		78.5	
Age ^b^ (years)	57.4	55.9		58.6		56.1		58.6		59.8		55.2	
Proportion of urban (1), agglomeration (2) and rural (3) residences ^c^	2.2	2.1		2.2		2.3		2.2		2.0		2.0	
Household size	2.4	2.3		2.3		2.5		2.5		2.4		2.5	
Number of children	0.34	0.36		0.33		0.38		0.39		0.26		0.28	
Education ^d^	4.6	4.6		4.4		4.9		4.3		4.6		4.9	
Employment ^e^	2.1	2.0		2.0		2.0		2.2		2.3		2.1	
Household income ^f^ (CHF)	7971	7980		7815		8625		7489		8014		8083	
Knowledge about nutrition (max. 20)	17.0	18.6	***	15.4	***	17.4		16.2	*	16.5		18.0	**
Body mass index (BMI)	24.6	23.1	***	26.2	***	24.4		24.8		25.2		23.6	*

^a^ The slightly higher proportion of women can be explained by the target group of the survey: people who are primarily responsible for grocery shopping in the household. In Switzerland, it is still more common for women than men to complete this chore. No official statistics exist for this target group. However, the proportion of men and women in Swiss society is almost equal (50.4% vs. 49.6% in 2020) [19]. ^b^ The adults in the household are primarily responsible for grocery shopping; hence the slightly higher average age (the average age of the Swiss adult population was 49.3 years in 2019) [20]. ^c^ As of 2019, 85% of the Swiss population lived in urban areas or agglomerations [21]. ^d^ 1—None, 2—Obligatory school and/or basic training, 3—Technical college, 4—Secondary school, 5—Higher vocational training, 6—Technical college, 7—University/Federal Institute of Technology (ETH). ^e^ 1—Fully employed, 2—Partly employed, 3—Unemployed/retired. ^f^ Exchange rate as of 1 October 2016: CHF 1 = CDN 1.35 = USD 1.03 = EUR 0.92. Significance levels: *** < 0.001, ** < 0.01, * < 0.05.

**Table 4:** Comparison of clusters with regard to other diet- and food consumption-related attitudes, preferences and behavioural variables.

	Ø	Cluster 1		Cluster 2		Cluster 3		Cluster 4		Cluster 5		Cluster 6	
Importance of the following aspects													
Eating a healthy diet	4.6	4.6		4.5	**	4.6		4.6		4.8		5.0	***
Consuming food that boosts one’s mood	4.5	4.3	*	4.4		4.4		4.4		4.6		4.8	**
Easy preparation of and access to food	4.1	3.9		4.0		4.0		4.0		4.1		4.2	
Good sensory quality of food	4.9	4.8		5.0		4.7		4.8		5.1	***	4.9	
Consuming natural foods	5.3	5.4		5.1		5.2		5.0	***	5.2		5.7	***
Consuming foods that help to control weight	4.0	3.6	***	3.8		4.2		4.0		4.2		4.1	
Consuming foods that I usually eat (as per habit)	3.8	3.2	***	4.2	***	3.5		3.8		4.2	***	3.6	
Consuming ethically correct foods	5.0	5.2		4.8	***	5.1		4.7	***	5.2		5.5	***
Low food prices and price promotions	4.1	3.7	*	4.3		4.0		4.1		4.3		3.9	
A good price/performance ratio	4.2	3.7	**	4.6	***	4.1		4.1		4.6	**	4.2	
Consuming organic food	4.4	4.7		4.0	***	4.5		3.9	***	4.4		5.3	***
Preparing meals yourself (cooking)	4.1	3.9		4.0		4.0		4.0		4.4	*	4.2	
Consuming Swiss food products	4.8	4.7		5.0		4.8		4.8		5.1	**	4.7	
Consuming regionally produced food products	4.6	4.6		4.3	**	4.8		4.3	***	4.8		5.0	***

For the items of all the scales, the agreement was queried as follows: 1—Do not agree at all, 6—Strongly agree. Significance levels: *** < 0.001, ** < 0.01, * < 0.05.

**Table 5:** Comparison of clusters with regard to respondents’ experiences with and consumption of meat and meat alternatives.

	Ø	Cluster 1		Cluster 2		Cluster 3		Cluster 4		Cluster 5		Cluster 6	
Have tried meat alternatives (%)	76	90		55		83		68		75		94	
Have bought meat alternatives (%)	57	77		27		65		51		45		82	
Consumed portions of meat, meat products & charcuterie weekly (100 g)	12.9	7.6	***	17.7	***	12.9		16.4	*	15.5	**	5.7	***
Consumed portions of meat alternatives (tofu, quorn, seitan or other meat alternatives) weekly (100 g)	1.3	1.7		0.2	***	0.7	***	0.7	**	0.5	***	4.2	***

Significance levels: *** < 0.001, ** < 0.01, * < 0.05.

**Table 6:** Comparison of clusters with regard to respondents’ agreement with arguments in favour of consuming meat alternatives.

	Ø	Cluster 1		Cluster 2		Cluster 3		Cluster 4		Cluster 5		Cluster 6	
What do you think are good reasons to consume meat alternatives?													
Fewer animals have to die.	4.3	4.5	***	3.1	***	5.0	***	3.8		4.3	***	5.5	
The population already eats too much protein anyway.	3.8	3.9		2.5	***	4.3	***	3.5	*	3.9		4.8	***
It is much more sustainable than eating meat.	3.8	4.6	***	2.5	***	4.7	***	3.2	***	3.6		4.8	***
There are many tasty meat alternatives.	3.3	4.2	***	2.2	***	3.6		3.0	**	2.6	***	4.3	***
The population already eats too much meat.	4.5	5.2	***	2.8	***	5.3	***	4.2	**	4.4		5.6	***

For the items of all the scales, the agreement was queried as follows: 1—Do not agree at all, 6—Strongly agree. Significance levels: *** < 0.001, ** < 0.01, * < 0.05.

**Table 7:** Comparison of clusters with regard to respondents’ agreement with arguments against consuming meat alternatives.

	Ø	Cluster 1		Cluster 2		Cluster 3		Cluster 4		Cluster 5		Cluster 6	
In your opinion, what are the reasons against consuming meat alternatives?													
It’s just not real meat.	4.1	3.0	***	5.3	***	4.0		4.5	**	4.7	***	3.1	***
Real men want real meat.	2.8	2.2	***	3.9	***	2.6		3.1		3.2	*	1.8	***
They just don’t taste the same (as real meat).	4.0	3.0	***	5.1	***	4.2		3.9		4.9	***	2.7	***
Meat is part of a balanced diet.	4.1	3.1	***	5.4	***	4.0		4.5	***	5.1	***	2.2	***
There are too few good meat alternatives.	3.9	3.1	***	4.5	***	4.3	**	3.9		4.4	**	3.2	***
Meat alternatives are too expensive.	3.5	3.0	**	3.7		3.6		3.6		4.0	**	2.9	**
There are enough tasty meatless dishes; meat alternatives are not necessary at all.	4.4	4.3		4.8	*	4.2		4.5		4.4		4.3	

For the items of all the scales, the agreement was queried as follows: 1—Do not agree at all, 6—Strongly agree. Significance levels: *** < 0.001, ** < 0.01, * < 0.05.
